# Putative acetylcholinesterase genes from a one-host tick species, the winter tick (*Dermacentor albipictus*)

**DOI:** 10.1186/s13071-025-07205-z

**Published:** 2026-02-14

**Authors:** Pia U. Olafson, Perot Saelao, David Luecke, Kimberly H. Lohmeyer, Kristie G. Schlechte, Kevin B. Temeyer

**Affiliations:** 1 Knipling-Bushland US Livestock Insects Research Laboratory, USDA-ARS, 2700 Fredericksburg Road, Kerrville, TX USA; 2 Livestock Arthropod Pests Research Unit, USDA-ARS, 2700 Fredericksburg Road, Kerrville, TX USA; 3 Veterinary Pest Genetics Research Unit, USDA-ARS, 2700 Fredericksburg Road, Kerrville, TX USA

**Keywords:** Ixodid, AChE, Genome

## Abstract

Tick control is heavily reliant on chemical acaricides; however, acaricide resistance and potentially toxic environmental effects underscore the need to develop new tick control strategies. Tick acetylcholinesterase is the target of organophosphate and carbamate acaricides, and the protein has been studied to develop novel anticholinesterase compounds that may exhibit significant improvements in arthropod specificity and mammalian safety. In support of these efforts, we mined the chromosome-level genome assemblies of *Dermacentor albipictus* and *Rhipicephalus microplus* to identify genes encoding acetylcholinesterase and evaluated transcript expression using publicly available *D. albipictus* transcriptome datasets. These one-host ixodid tick species are of significance to wildlife and livestock health, and genomic comparisons between them are highly relevant given their shared lifecycle. Our *in silico* search identified 73 and 52 putative AChE-like protein sequences in the *D. albipictus* and *R. microplus* genomes, respectively, and these encode conserved domains and features consistent with enzymatically active cholinesterases. AChE1 is one of the major AChEs involved in neuronal transmission in insects, and a clade of AChE1-like sequences that included representatives from *D. albipictus* and *R. microplus* was observed, including the BmAChE1 protein that has been enzymatically characterized. Alternative splicing and gene duplication events were evident, including a conserved AChE gene duplication event that appears across five tick species, indicating a role for these processes in AChE diversification. Different expression patterns of *AChE* were observed, with indication that its expression was coincident with the expression of receptors for acetylcholine, the substrate for AChE. The notable number of AChE-encoding genes supports the diversification and specialization of this gene family in one-host tick species, emphasizing the complex role that AChEs play in tick physiology and development.

**Graphical Abstract:**

**Figure:**
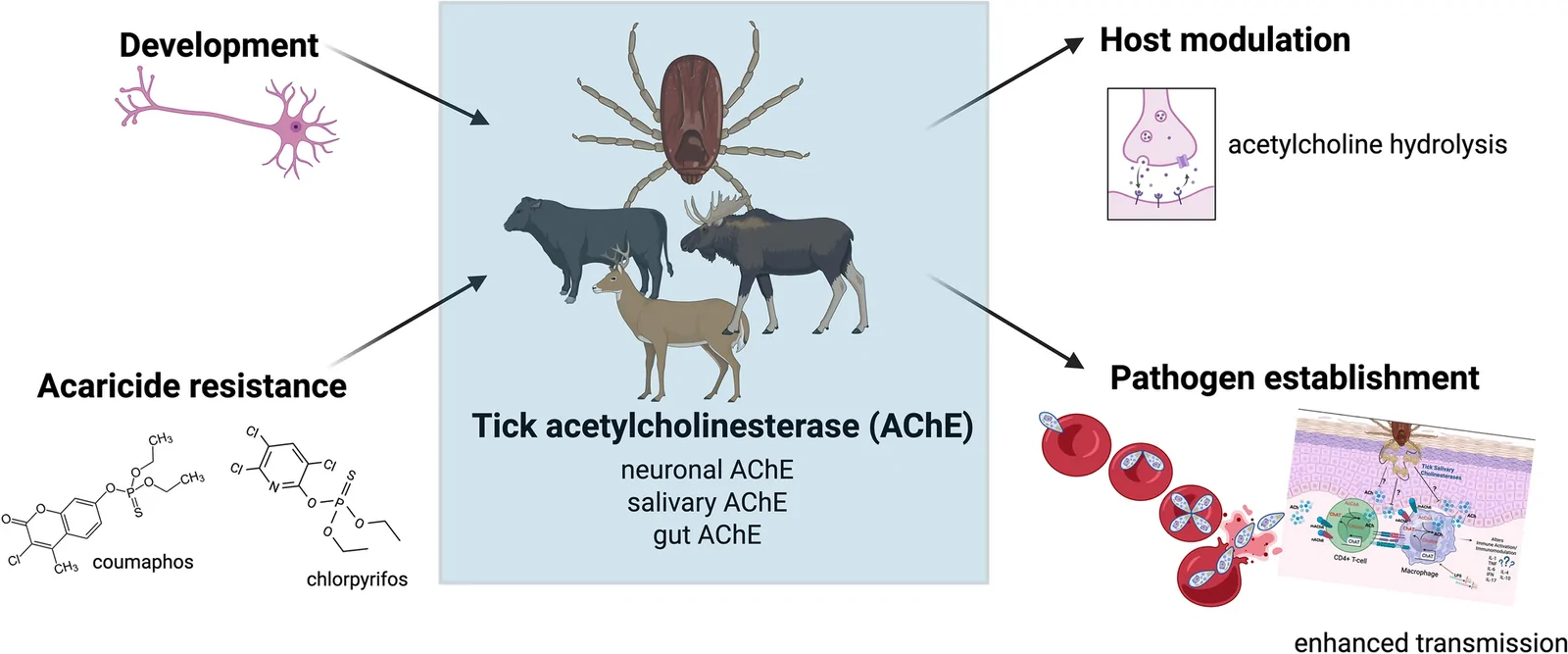

**Supplementary Information:**

The online version contains supplementary material available at https://doi.org/10.1186/s13071-025-07205-z.

Ticks transmit disease-causing pathogens that are of consequence to human and animal health. Integrated tick management strategies include habitat modification, repellents, and biological control methods, yet tick control, particularly on livestock, has largely relied on chemical acaricides [1, 2]. The widespread use of acaricides has led to the development of tick resistance to these chemicals. Most significantly, this has been reported worldwide for the southern cattle tick, *Rhipicephalus microplus*, that transmits the pathogens causing bovine babesiosis and bovine anaplasmosis and is the most important tick species affecting livestock globally [3]*.* The one-host biology of this species leads to increased exposure of all its lifestages to acaricide pressure, increasing the likelihood of resistance development [4].

Acetylcholinesterase (AChE) is an enzyme that has a critical role in the nervous system, as it hydrolyzes the neurotransmitter acetylcholine to terminate nerve transmission [5]. Organophosphate and carbamate compounds were first developed as nerve agents that were subsequently commercialized as pesticides because of their AChE inhibition properties, resulting in excessive acetylcholine buildup that was lethal towards arthropod pests [6]. Recently, AChE has been the intended target for the development of novel arthropod-specific acaricides with the goal of identifying inhibitors that have limited toxicity towards human and animal hosts [7–9]. In support of these efforts, it is critical to identify and enzymatically characterize AChEs from tick species of importance.

Ixodid tick and arachnid genomes comprise multiple genes encoding AChEs [10, 11], which is in contrast to many insects that have only one or two genes [12]. Three different AChEs from *R. microplus* (BmAChE1, BmAChE2, and BmAChE3) were extensively characterized by Temeyer et al. [13], confirming differences in their biological, genetic, and biochemical properties, including evidence for their redundancy as a compensatory mechanism for survival [14]. Within Ixodidae, few tick species exhibit a one-host tick lifecycle. To enable genomic comparisons between representative one-host species, our group recently produced chromosome-level genome assemblies for *R. microplus* [15] and for the winter tick, *Dermacentor albipictus*, a one-host, ixodid tick that is widely distributed across North America [16]. *Dermacentor albipictus* predominantly infest wild and domestic ungulates, particularly moose, elk, caribou, and deer in extremely high numbers, sometimes resulting in death [17, 18]. This species is a reported vector of *Anaplasma marginale* and *Babesia duncani*, and there is evidence that it harbors *Rickettsia felis* [19–21]. Cattle are readily parasitized by *D. albipictus* [22], and acaricidal pressure may have similar consequences on this species as it does for *R. microplus*. Here, we mined the *D. albipictus* genome assembly for genes encoding putative AChEs, and we evaluated the phylogenetic relationship of these sequences between these one-host species. We highlight a remarkable number of tick proteins in the AChE gene family and describe evidence for alternative splicing and gene duplication that underscore the complexity of tick acetylcholinesterases.

The chromosome-level genomes of *R. microplus* (USDA_Rmic, GCA_043290135) and *D. albipictus* (USDA_Dalb.pri_finalv2, GCA_038994185) were annotated by the NCBI using the Eukaryotic Genome Annotation Pipeline. A gene list was populated by downloading those sequences annotated as “acetylcholinesterase,” then evaluating the protein sequences using the NCBI Conserved Domain analysis tool [23]. Three well-characterized AChE genes from *R. microplus* (GenBank Accessions USH09479 (BmAChE1), AAC18857 (BmAChE2), and AAP92139 (BmAChE3)), an *Amblyomma americanum* cholinesterase (WDY73567; [24]), and an *R. microplus* carboxylesterase (WMQ58240; [25]) were used as positive controls for conserved domains since their enzymatic activity has been confirmed experimentally. As such, amino acid sequences mined from the genomes were included in the analysis if they encoded the “Ab (alpha–beta) hydrolase superfamily” (cl21494), “Carboxylesterase (COesterase)” (pfam00135), “Esterase_lipase” (cd00312), and “PnbA” (carboxylesterase type B; COG2272) domains, as well as their encoding “catalytic triad” and “substrate binding” features. Putative signal peptides and subcellular localizations were predicted using SignalP 6.0 and DeepLoc, respectively [26]. A separate gene list was populated by searching the *D. albipictus* genome for sequences annotated as “choline acyltransferase” or “acetylcholine receptor.” These, too, were submitted to the NCBI Conserved Domain analysis tool for identification of domains and features. Putative nicotinic or muscarinic acetylcholine receptors were included in the analysis if they encoded “ligand gated ion channel, extracellular domain” (cl28912), “ligand-gated ion channel, transmembrane” (cd19051), “7-transmembrane muscarinic acetylcholine receptor” (cd15049; DM1 and M1 like), or “neurotransmitter-gated ion-channel ligand binding domain” (cl47783) conserved domains. Further, choline acyltransferase sequences were included if they encoded a “Choline/Carnitine o-acyltransferase” (cl08288) domain.

The AChE amino acid sequences were aligned using MUSCLE v5.3 with the -super5 option [27], and *Drosophila melanogaster* esterase (AAP21002) was used as an outgroup. All sequences are available in Supplementary File S1. A phylogenetic tree was then inferred from this alignment using IQ-Tree v3.0.1 with ModelFinder, SH-aLRT test (–alrt 1000), and ultrafast bootstrap (–B 1000) options [28–30]. As part of the NCBI annotation pipeline, gene models encoding each protein sequence were available in the NCBI Sequence Viewer. These were examined for each transcript to validate exon–intron structure and identify evidence of alternative splicing, and these instances were recorded (Supplementary File S1). Based on phylogenetic inference, a set of duplicated AChE genes in the *D. albipictus* and *R. microplus* genomes occupied the same clade, possibly suggestive of a conserved duplication. To identify whether this may be present in other tick species, the BLAST algorithm was used to search chromosome-level genome assemblies of *Dermacentor variabilis* (American dog tick; ASM5094787v1, NC_134572), *Dermacentor andersoni* (Rocky Mountain wood tick; qqDerAnde1, NC_092818.1), and *Rhipicephalus sanguineus* (brown dog tick; BIME_Rsan_1.4, NC_051180) for the presence of sequences homologous to the duplicated set. Transcript expression profiles were obtained for sequences on both gene lists using available *D. albipictus* RNA-seq datasets [31] representing unfed, newly molted adult females (uf_Female), unfed newly molted adult males (uf_Male), 0–6 h embryos (embr_06), ovaries from preoviposition females (ov_preov), and reproductive systems from fed, unmated males (MRS_fum). These reads were quantified at the transcript level and quasi-mapped to the reference GCA_038994185 using Salmon [32]. Bias models were enabled, and sample-specific fragment length distributions were estimated during processing. Salmon produced raw transcript per million counts (TPMs), while transcript estimates to genes were performed with tximport in R [33] using a transcript-to-gene map. Gene-level data were normalized (size-factor normalization) and processed using DESeq2 [34]. Visualization of normalized counts were transformed to row-wise z-scores calculated through R’s heatmap function. Data are accessible in SRA Accessions SRX24226854-SRX24226858. The inferred AChE phylogenetic tree was visualized and annotated using ggtree [35], and heat maps were produced to represent the expression profiles for select genes using ggplot2 in R [36].

A query of the *D. albipictus* and *R. microplus* genomes identified 156 and 91 protein-coding sequences, respectively, that were annotated as “acetylcholinesterase” or “acetylcholinesterase-like.” Identical proteins (33 in *D. albipictus*, 17 in *R. microplus*), resulting from sequence variation in the 5′ or 3′ untranslated regions of a locus, were removed. We further curated this dataset to eliminate proteins that lacked features consistent with a functional AChE enzyme. The alpha–beta hydrolase protein superfamily, to which acetylcholinesterase belongs, comprises enzymes that act on carboxylic esters, and these reactions involve a triad of amino acid residues (“catalytic triad”) that are essential for catalyzing enzymatic activity. Specifically, AChE is a serine hydrolase with a distinct catalytic triad comprising serine, glutamic acid, and histidine, which are required for the hydrolysis of acetylcholine (Supplementary File S1; [37]). As such, sequences were included if they encoded the catalytic triad Ser/Glu/His residues that define a functional AChE enzyme. This resulted in the further removal of 50 *D. albipictus* and 22 *R. microplus* proteins. We confirmed the presence of BmAChE1 and BmAChE2 sequences in this dataset, while BmAChE3 was identified within locus LOC119171776, a fusion of two gene models, and was added to the gene list. After curation, the dataset comprised 73 *D. albipictus* and 52 *R. microplus* proteins, and the phylogenetic relationship of these sequences was inferred (Fig. 1, Supplementary File S1), reflecting highly diverse AChEs in these one-host tick species. Of note is the distinct clade of AChE1-like proteins (Fig. 1, purple box) with representative sequences obtained from GenBank for *Tetranychus urticae* (two-spotted spider mite) and *Blatella germanica* (German cockroach, insect *ace-1* and *ace-2*). In insects, AChE1 is primarily associated with neuronal functions, and it has the closest relationship to the neuronal AChE in vertebrates [12]. The *R. microplus* BmAChE1 and *A. americanum* cholinesterase are also present within this clade, and their enzymatic activity and substrate preference for acetylcholine has been described [13, 24]. The current analysis identified five homologous AChE1-like sequences in *D. albipictus*, and it also highlighted two additional paralogs in *R. microplus* that are AChE1-like. Proteins within the sister clade to AChE-1 like proteins exhibit all hallmarks of AChE and are labeled “non-AChE1” (Fig. 1).

**Fig. 1:**
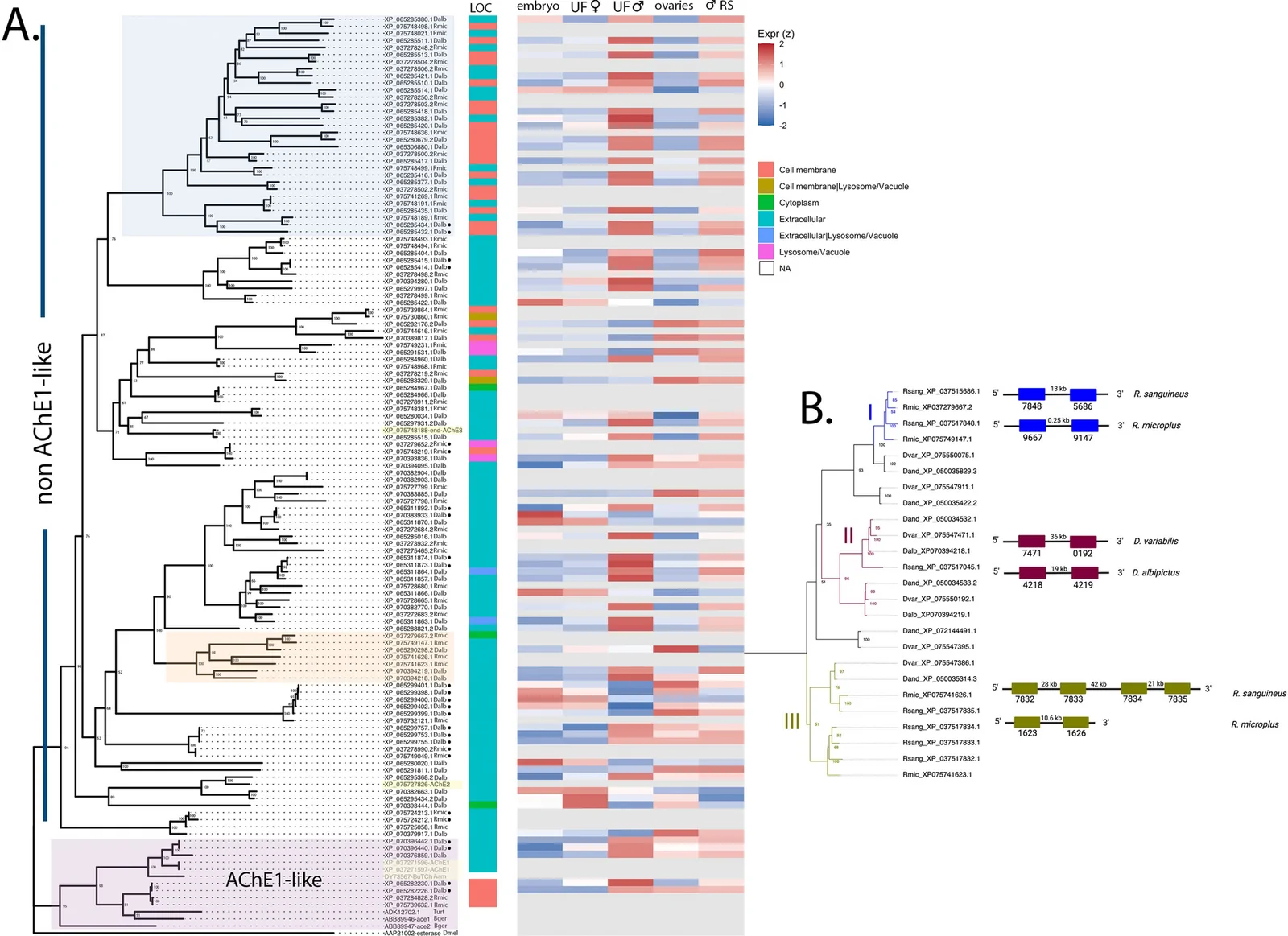
**A** Inferred phylogeny of putative acetylcholinesterase (AChE) proteins identified from the chromosome-level genome assemblies of the winter tick, *Dermacentor albipictus* (Dalb), and the cattle tick, *Rhipicephalus microplus* (Rmic). Amino acid sequences were aligned using MUSCLE, and the phylogenetic tree was inferred using IQTree (best-fit substitution model, branch support assessed with 1000 replicates of UFBoot bootstrap approximation; boostrap percentage reflected). A *Drosophila melanogaster* (Dmel) esterase (AAP21002) was used as an outgroup, and AChE sequences from *Tetranychus urticae* (Turt; ADK12702) and *Blatella germanica* (Bger; ABB89946, ABB89947) are identified. All sequences are available in Supplementary File S1. Predicted cell localizations are represented by a vertical bar (see legend in figure), and a heat map of corresponding *D. albipictus AChE* transcripts mined from existing RNA-seq data is presented [31]. Filled circles on taxa labels identify those isoforms produced by alternative splicing (Supplementary File S1) and taxa highlighted in yellow identify those *R. microplus* and *Amblyomma americanum* (Aam) sequences that have experimental evidence of enzymatic activity towards acetylthiocholine, the preferred substrate for AChE. A purple box identifies a clade of AChE1-like sequences, and a sister clade comprising non-AChE1-like sequences is labeled. A blue box identifies a clade of non-AChE1-like sequences that are predicted to be localized to the cell membrane. An orange box identifies a putatively conserved AChE gene duplication (see text). **B** Inferred phylogeny of AChE proteins from *Rhipicephalus sanguineus* (Rsang), *D. variabilis* (Dvar), and *D. andersoni* (Dand) that are homologous to duplicated AChEs from *D. albipictus* and *R. microplus*. Clades I, II, and III identify duplicated sets (see text), and representative gene organizations and distances between duplications within the respective genomes are depicted. Filled circles on taxa identify those isoforms produced by alternative splicing

Cholinesterases have evolved to serve different functions, which can account for the multiple genes that code for AChE in invertebrate genomes [10, 11]. Alternative splicing is one mechanism that can contribute to an increase in protein diversity and alternative tissue expression [38, 39], and there is evidence of this occurring in the genes encoding AChE in *D. albipictus* and *R. microplus*. Specifically, gene models for 10 loci in *D. albipictus* are putatively alternatively spliced to produce 25 AChE-like isoforms, with variability in the first exon being encountered most frequently (Supplementary File S1). In *R. microplus,* different exon usage at four loci produced eight isoforms. Furthermore, alternative splicing of tick AChEs has been reported from *R. australis* (nee *R. microplus*), *R. sanguineus*, and *R. annulatus* [40, 41]. In the latter, Temeyer et al. [42] observed comparable biochemical properties between BmAChE1 and three alternatively spliced AChE1-like isoforms from *R. annulatus*, further supporting a compensatory role for these isoforms. Gene duplication is also a mechanism that can contribute to the emergence of genes with diverse functions, and we observed evidence of this at two sites within the *D. albipictus* genome. In these instances, the genes were located within 19 kb and 11.8 kb of each other on chromosomes 4 and 10, respectively, and they encoded AChE proteins that were 60% and 90% identical (Supplementary File S1), respectively. Similarly, gene duplication of putative AChEs was evident in *R. microplus* with genes located 0.25 kb and 21 kb apart and 10.6 kb apart on chromosome 4 and an unplaced scaffold, respectively, exhibiting 83%, 96%, and 57% amino acid identity (Supplementary File S1). One *D. albipictus* gene duplication event and two *R. microplus* events are on the same branch (Fig. 1, orange box), suggesting the duplications may be conserved. To address this, homologues of these representative duplications were identified from the chromosome-level genome assemblies of *D. variabilis*, *D. andersoni*, and *R. sanguineus*. The homologues encoded hallmarks of AChEs, as confirmed using the Conserved Domain database, and they were used in phylogenetic comparison with the *D. albipictus* and *R. microplus* duplications (Fig. 1B). Clade I includes two genes encoding *R. sanguineus* AChEs that are 13 kb apart and 83% identical, sharing a lineage with an *R. microplus* duplication. In clade II, which includes the *D. albipictus* duplication, two genes encoding *D. variabilis* AChE are separated by 36 kb and are 61% identical. In addition, this clade included two *D. andersoni* AChE isoforms (XP_050034532 and XP_050034533) resulting from alternative splicing at a single locus (LOC126531156). Lastly, in clade III, which includes an *R. microplus* duplication, four genes encoding *R. sanguineus* AChE are arranged in tandem, separated by 21–42 kb, and exhibit 58–80% identity (Supplementary File S3). Collectively, these results support conservation of this duplication event across several tick species.

*In silico* predictions of protein localization and membrane associations using DeepLoc 2.1 identified 52 *D. albipictus* and 36 *R. microplus* putative AChE sequences as extracellular (Fig. 1), all of which were predicted to be soluble proteins (Supplementary File S2). Within this set, there were two *D. albipictus* AChEs that had comparable probabilities of extracellular or lyososomal/vacuole localization, and these are reflected in Fig. 1. Of the collective 30 sequences predicted to be localized to the cell membrane, 23 had signals of being lipid anchored and 7 of being transmembrane (Supplementary File S2). As above, there were two sequences with similar probabilities of cell membrane or lysosomal/vacuole localization (Fig. 1). These differences in localization are consistent with the varied tissue distribution and functions of AChEs. Interestingly, the clades comprising *R. microplus* AChEs for which enzymatic activity has been validated are not predominantly extracellular/soluble (Fig. 1, taxa highlighted in yellow). For example, the lineage where *R. microplus* AChE3 resides includes proteins predicted to be extracellular, as well as localized to the cell membrane and/or lysosome/vacuoles. Further, within the AChE1-like lineage, there are sister clades comprising paralogs predicted to be either extracellular (includes BmAChE1) or cell membrane-bound (Fig. 1). Of the 30 AChEs predicted to be localized to the cell membrane, more than half (*n* = 19) are on a separate clade of non-AChE1 like proteins (Fig. 1, blue box). The *D. albipictus* genes within this clade are predominantly expressed in newly molted males and reproductive systems of fed males, with limited expression detected in embryos or females/female tissues (Fig. 1). The differential expression pattern of *AChEs* in all tissues and lifestages, though, is evident in genes outside this clade. Choline acyltransferase (ChAT) is an essential enzyme that catalyzes the transfer of an acetyl group from coenzyme acetyl-CoA to choline, yielding acetylcholine. Acetylcholine receptors (AChR) bind acetylcholine and mediate physiological responses that differ in speed and mechanism, which is reflected in the two types of AChRs, nicotinic (nAChR) and muscarinic (mAChR)  [43]. The nAChRs are ligand-gated ion channels that enable fast synaptic transmission at neuromuscular junctions, while mAChRs are G-protein coupled receptors that initiate second messenger pathways to regulate arthropod physiology. In the *D. albipictus* RNA-seq, three *ChAT* transcripts were identified, as were 25 *nAChR* and three *mAChRs* (Supplementary File S4). Given the expression profile of *D. albipictus AChE*s, we examined whether there was any correlation with the expression of transcripts encoding ChAT and AChRs (Fig. 2). Interestingly, a cluster of transcripts had a biased expression in embryos and newly molted adult females, and the cluster included one *ChAT*, six *nAChR*, and all three *mAChRs*. Conversely, the other two *ChAT* transcripts and one *nAChR* exhibited biased expression in preoviposition ovaries and male reproductive systems. Coincident with biased *AChE* expression in newly molted adult males, 17 *nAChRs* had a comparable expression pattern and were elevated relative to the other tissues and lifestages examined, possibly reflecting an increased need for removal of the neurotransmitter. However, these data represent only one snapshot in time and lack information from neuronal tissues (i.e., tick synganglia). In *R. microplus*, the three verified AChEs are all expressed in synganglia, as well as other non-neuronal tissues [10]. Given the complex expression network underlying this pathway, these analyses would be strengthened by additional RNA-seq datasets for *D. albipictus*.

**Fig. 2:**
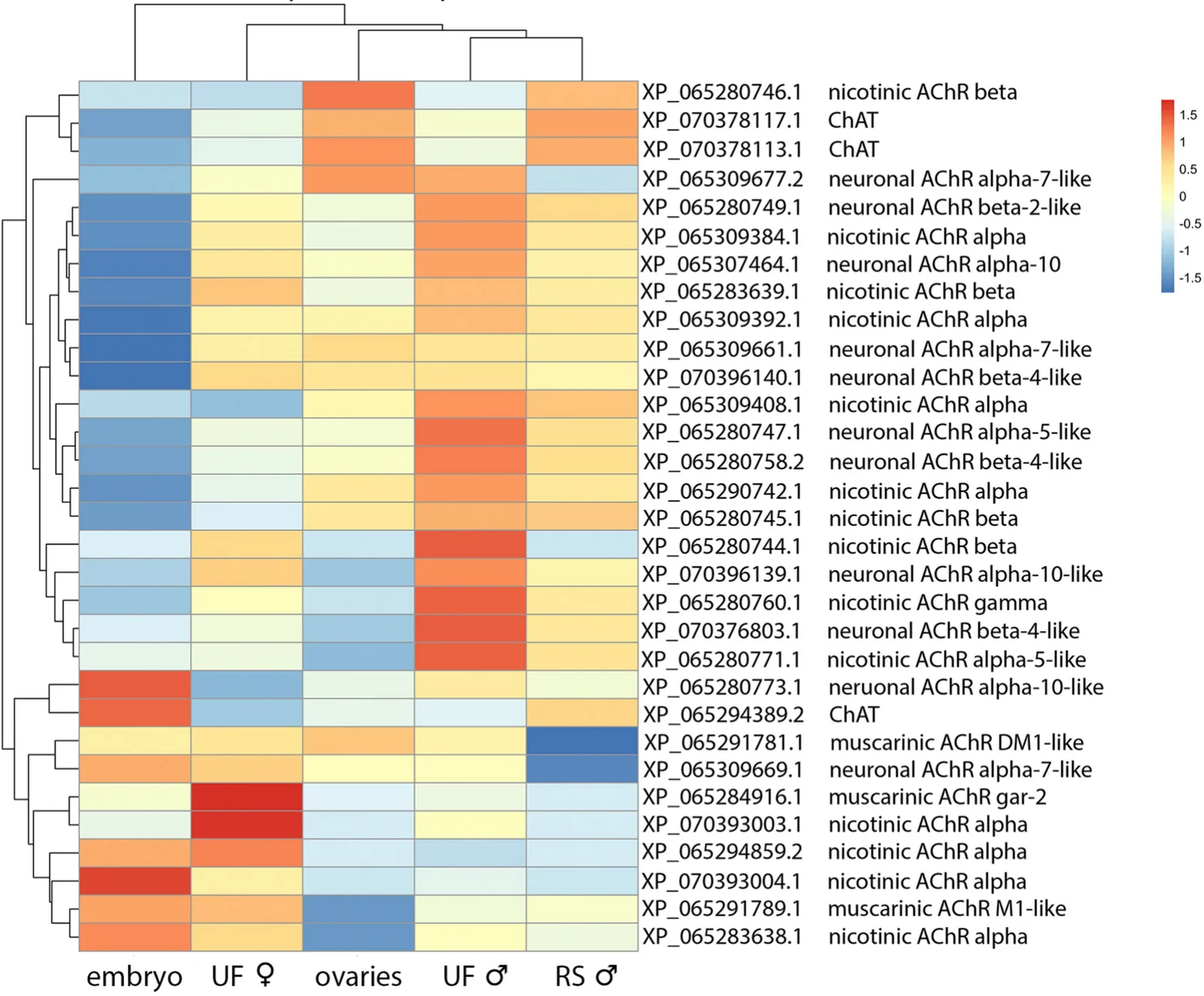
Heatmap representing the transcript expression profile of choline acyltransferase (*ChAT*) and acetylcholine receptors (*AChR*), either nicotinic (nAChR, neuronal AChR) or muscarinic, in available RNA-seq datasets from *Dermacentor albipictus* [31]. Using a heatmap, genes with similar expression patterns are clustered and normalized counts were transformed to row-wise z-scores

The expression of putatively cell membrane-bound AChE in non-neuronal reproductive tissues supports involvement in nonclassical roles, such as cell differentiation or apoptosis, as suggested by Silman [44]. These nonenzymatic roles further include AChE interaction with proteins to enable cell adhesion that supports cell signaling [45]. Rapid cell division and differentiation are occurring in reproductive tissues and, based on the expression data here, AChEs may have a role during these processes. AChEs have also been implicated in regulating expression of arthropod immune response pathways and stress tolerance [46–48], which are critical defenses for newly molted adult ticks. Of curiosity are the 50 *D. albipictus* and 22 *R. microplus* proteins that were annotated as acetylcholinesterase, but excluded from the gene list because they did not encode the Ser/Glu/His catalytic triad. These were predominantly predicted to be localized to the cell membrane and exhibited a transcript expression profile biased towards reproductive tissues (Olafson, unpublished), suggesting the importance of enzymes in the alpha-hydrolase superfamily to these one-host tick species.

Chromosome-level genome assemblies enable comparative analysis of gene families, and the increased numbers of AChEs identified from one-host ticks suggests diversification and specialization of this gene family. Indeed, recent evidence indicates AChE is secreted in tick saliva, possibly having a role in both pathogen transmission and host immunomodulation [49, 50]. Cholinergic systems play a key role in physiology and metabolism, likely reflecting the ancient emergence of acetylcholine [10]. Our *in silico* analysis identifies diverged, tick-specific AChE paralogs as candidate targets for new acaricide development, supporting additional biochemical characterization to verify enzymatic or nonenzymatic activities and define their roles in tick biology.

## Supplementary Information

Supplementary Material 1: File S1. Putative acetylcholinesterase proteins from the genome of the winter tick, Dermacentor albipictus, and the southern cattle tick, Ripicephalus microplus.

Supplementary Material 2: File S2. Results from DeepLoc analysis of putative AChEs from Dermacentor albipictus and Rhipicephalus microplus.

Supplementary Material 3: File S3. AChE percent identity matrix for representative gene duplications in Figure 1B.

Supplementary Material 4: File S4. Putative choline acyltransferase (ChAT) and nicotinic or muscarinic acetylcholine receptor (AchR) transcripts from the genome of the winter tick, Dermacentor albipictus.

## Data Availability

The datasets analyzed during the current study are publicly available in NCBI, to include the chromosome-level genomes of *R. microplus* (USDA_Rmic, GCA_043290135) and *D. albipictus* (USDA_Dalb.pri_finalv2, GCA_038994185) and the *D. albipictus* RNA-seq datasets (Accessions SRX24226854-SRX24226858).

## References

[1] Tiffin HS, Rajotte EG, Sakamoto JM, Machtinger ET. Tick control in a connected world: challenges, solutions, and public policy from a United States border perspective. Trop Med Infect Dis. 2022. 10.3390/tropicalmed7110388. PMC9695313 36422939

[2] Stafford KC III, Williams SC, Molaei G. Integrated pest management in controlling ticks and tick-associated diseases. J Integr Pest Manag. 2017;28:1–7. 10.1093/jipm/pmx018.

[3] Klafke GM, Golo PS, Monteiro CMO, Costa-Junior LM, Reck J. Brazil’s battle against *Rhipicephalus* (*Boophilus*) *microplus* ticks: current strategies and future directions. Rev Bras Parasitol Vet. 2024;33:e001423. 10.1590/S1984-29612024026. PMC11253825 38922203

[4] Thomas DB, Klafke G, Busch JD, Olafson PU, Miller RA, Mosqueda J, et al. Tracking the increase of acaricide resistance in an invasive population of cattle fever ticks (Acari: Ixodidae) and implementation of real-time PCR assays to rapidly genotype resistance mutations. Ann Entomol Soc Am. 2020;113:298–309. 10.1093/aesa/saz053.

[5] Casida JE, Durkin KA. Neuroactive insecticides: targets, selectivity, resistance, and secondary effects. Annu Rev Entomol. 2013;58:99–117. 10.1146/annurev-ento-120811-153645. 23317040

[6] Casida JE, Durkin KA. Anticholinesterase insecticide retrospective. Chem Biol Interact. 2013;203:221–5. 10.1016/j.cbi.2012.08.002. PMC3572339 22926007

[7] Carlier PR, Bloomquist JR, Totrov M, Li J. Discovery of species-selective and resistance-breaking anticholinesterase insecticides for the malaria mosquito. Curr Med Chem. 2017;24:2946–58. 10.2174/0929867324666170206130024. PMC5755385 28176636

[8] Li C, Cao Y, Yang J, Li M, Li B, Bu C. Acetylcholinesterase target sites for developing environmentally friendly insecticides against *Tetranychus urticae* (Acari: Tetranychidae). Exp Appl Acarol. 2021;84:419–31. 10.1007/s10493-021-00624-4. 33914192

[9] Swale DR, Tong F, Temeyer KB, Li A, Lam PC, Totrov MM, et al. Inhibitor profile of bis(n)-tacrines and N-methylcarbamates on acetylcholinesterase from *Rhipicephalus* (*Boophilus*) *microplus* and *Phlebotomus papatasi*. Pestic Biochem Physiol. 2013;106:10.1016/j.pestbp.2013.03.005. 10.1016/j.pestbp.2013.03.005. PMC3811934 24187393

[10] Temeyer KB. Molecular biology of tick acetylcholinesterases. Front Biosci. 2018;23:1320–37. 10.2741/4646. 28930602

[11] Liu H, Jang J, French AS, Torkkeli PH. Sequence analysis, homology modeling, tissue expression, and potential functions of seven putative acetylcholinesterases in the spider *Cupiennius salei*. Eur J Neurosci. 2024;60:5785–811. 10.1111/ejn.16524. 39230060

[12] Kim YH, Lee SH. Which acetylcholinesterase functions as the main catalytic enzyme in the Class Insecta? Insect Biochem Mol Biol. 2013;43:47–53. 10.1016/j.ibmb.2012.11.004. 23168079

[13] Temeyer KB, Pruett JH, Olafson PU. Baculovirus expression, biochemical characterization and organophosphate sensitivity of rBmAChE1, rBmAChE2, and rBmAChE3 of *Rhipicephalus* (*Boophilus*) *microplus*. Vet Parasitol. 2010;172:114–21. 10.1016/j.vetpar.2010.04.016. 20451328

[14] Temeyer KB, Tijerina M, Davey RB, Olafson PU. Genetic factors potentially reducing fitness cost of organophosphate-insensitive acetylcholinesterase(s) in *Rhipicephalus* (*Boophilus*) *microplus* (Acari: Ixodidae). Zoosymposia. 2011;6:260–6. 10.11646/zoosymposia.6.1.35.

[15] Tidwell JP, Bendele KG, Bodine D, Holmes VR, Johnston JS, Saelao P, et al. Identifying the sex chromosome and sex determination genes in the cattle tick, *Rhipicephalus* (*Boophilus*) microplus. G3 (Bethesda). 2024;14:jkae234. 10.1093/g3journal/jkae234. PMC11631522 39344017

[16] Chenery ES, Harms NJ, Fenton H, Mandrak NE, Molnár PK. Revealing large‐scale parasite ranges: an integrated spatiotemporal database and multisource analysis of the winter tick. Ecosphere. 2023;14:e4376. 10.1002/ecs2.4376.

[17] Machtinger ET, Springer HR, Brown JE, Olafson PU. Sudden mortality in captive white-tailed deer with atypical infestation of winter tick. J Med Entomol. 2021;58:1962–5. 10.1093/jme/tjab043. 33764454

[18] Saleh MN, Ramos RAN, Verocai GG, Monahan CF, Goss R, Needle DB. Apparent fatal winter tick (*Dermacentor albipictus*) infestation in captive reindeer (*Rangifer tarandus*). Int J Parasitol Parasites Wildl. 2024;23:100900. 10.1016/j.ijppaw.2023.100900. PMC10808900 38274347

[19] Hairgrove TB, Craig TM, Budke CM, Rodgers SJ, Gill RJ. Seroprevalence of *Anaplasma marginale* in Texas cattle. Prev Vet Med. 2014;116:188–92. 10.1016/j.prevetmed.2014.05.008. 24931130

[20] Swei A, O’Connor KE, Couper LI, Thekkiniath J, Conrad PA, Padgett KA, et al. Evidence for transmission of the zoonotic apicomplexan parasite *Babesia duncani* by the tick *Dermacentor albipictus*. Int J Parasitol. 2019;49:95–103. 10.1016/j.ijpara.2018.07.002. PMC10016146 30367862

[21] Goddard J, Snellgrove AN, Sims S, Robbins RG, Varnado WC. Molecular detection of *Rickettsia felis* in *Dermacentor albipictus* from Mississippi. J Med Entomol. 2025. 10.1093/jme/tjaf129. 40971700

[22] Teel PD, Fuchs TW, Huston JE, Longnecker MT, Pickel SL. Effects of sequential infestations of *Dermacentor albipictus* and *Amblyomma americanum* (Acari: Ixodidae) on overwintering beef cows in west-central Texas. J Med Entomol. 1990;27:632–41. 10.1093/jmedent/27.4.632. 2388237

[23] Lu S, Wang J, Chitsaz F, Derbyshire MK, Geer RC, Gonzales NR, et al. CDD/SPARCLE: the conserved domain database in 2020. Nucleic Acids Res. 2020;48:D265–8. 10.1093/nar/gkz991. PMC6943070 31777944

[24] Temeyer KB, Schlechte KG, Gross AD, Lohmeyer KH. Identification, baculoviral expression, and biochemical characterization of a novel cholinesterase of *Amblyomma americanum* (Acari: Ixodidae). Int J Mol Sci. 2023. 10.3390/ijms24097681. PMC10178864 37175388

[25] Labuschagne M. Cloning, recombinant expression, and characterization of a *Rhipicephalus microplus* carboxylesterase. Gates Open Res. 2024;8:87. 10.12688/gatesopenres.16245.1. PMC11813172 39936143

[26] Nielsen H. Practical applications of language models in protein sorting prediction: SignalP 6.0, DeepLoc 2.1, and DeepLocPro 1.0. In: Kc DB, editor. Large Language Models (LLMs) in protein bioinformatics. New York: Springer, US; 2025. p. 153–75. 10.1007/978-1-0716-4623-6_10 40601257

[27] Edgar RC. Muscle5: high-accuracy alignment ensembles enable unbiased assessments of sequence homology and phylogeny. Nat Commun. 2022;13:6968. 10.1038/s41467-022-34630-w. PMC9664440 36379955

[28] Hoang DT, Chernomor O, von Haeseler A, Minh BQ, Vinh LS. UFBoot2: improving the ultrafast bootstrap approximation. Mol Biol Evol. 2018;35:518–22. 10.1093/molbev/msx281. PMC5850222 29077904

[29] Kalyaanamoorthy S, Minh BQ, Wong TKF, von Haeseler A, Jermiin LS. ModelFinder: fast model selection for accurate phylogenetic estimates. Nat Methods. 2017;14:587–9. 10.1038/nmeth.4285. PMC5453245 28481363

[30] Nguyen LT, Schmidt HA, von Haeseler A, Minh BQ. IQ-TREE: a fast and effective stochastic algorithm for estimating maximum-likelihood phylogenies. Mol Biol Evol. 2015;32:268–74. 10.1093/molbev/msu300. PMC4271533 25371430

[31] Edwards RTM, Antoshechkin I, Hill E, Perry MW, Olafson PU, Saelao P, et al. First transcriptome analysis of the winter tick (*Dermacentor albipictus*) reveals sex-specific expression patterns and potential targets for genetic control. G3 Genes Genomes Genet. 2025. 10.1093/g3journal/jkaf116. PMC12341929 40408551

[32] Patro R, Duggal G, Love MI, Irizarry RA, Kingsford C. Salmon provides fast and bias-aware quantification of transcript expression. Nat Methods. 2017;14:417–9. 10.1038/nmeth.4197. PMC5600148 28263959

[33] Soneson C, Love MI, Robinson MD. Differential analyses for RNA-seq: transcript-level estimates improve gene-level inferences. F1000Res. 2015;4:1521. 10.12688/f1000research.7563.2. PMC4712774 26925227

[34] Love MI, Huber W, Anders S. Moderated estimation of fold change and dispersion for RNA-seq data with DESeq2. Genome Biol. 2014;15:550. 10.1186/s13059-014-0550-8. PMC4302049 25516281

[35] Yu G, Smith DK, Zhu H, Guan Y, Lam TT-Y. Ggtree: an r package for visualization and annotation of phylogenetic trees with their covariates and other associated data. Methods Ecol Evol. 2017;8:28–36. 10.1111/2041-210X.12628.

[36] Wickham H. ggplot2: elegant graphics for data analysis. New York: Springer-Verlag; 2016.

[37] Hung LW, Sanbonmatsu KY, Williams RF, Chen JC. Acetylcholinesterase: structure, dynamics, and interactions with organophosphorus compounds. Protein Sci. 2025;34:e70297. 10.1002/pro.70297. PMC12447245 40970461

[38] Mohr C, Hartmann B. Alternative splicing in Drosophila neuronal development. J Neurogenet. 2014;28:199–215. 10.3109/01677063.2014.936437. 24957133

[39] Venables JP, Tazi J, Juge F. Regulated functional alternative splicing in Drosophila. Nucleic Acids Res. 2012;40:1–10. 10.1093/nar/gkr648. PMC3245913 21908400

[40] Baxter GD, Barker SC. Acetylcholinesterase cDNA of the cattle tick, *Boophilus microplus*: characterisation and role in organophosphate resistance. Insect Biochem Mol Biol. 1998;28:581–9. 10.1016/s0965-1748(98)00034-4. 9753768

[41] Xu G, Fang QQ, Keirans JE, Durden LA. Cloning and sequencing of putative acetylcholinesterase cDNAs from the American dog tick, *Dermacentor variabilis*, and the brown dog tick, *Rhipicephalus sanguineus* (Acari: Ixodidae). J Med Entomol. 2003;40:890–6. 10.1603/0022-2585-40.6.890. 14765667

[42] Temeyer KB, Schlechte KG, Saelao P, Olafson PU, Tidwell JP. Acetylcholinesterase 1 of the cattle tick, *Rhipicephalus* (*Boophilus*) *microplus*. J Med Entomol (Under Rev). 2025;106:118–23. 10.1093/jme/tjag025 41736573

[43] Boussaine K, Chartier P, Cartereau A, Auguin D, Taillebois E, Thany SH. New insight into the tick neuronal nicotinic acetylcholine receptors: from molecular to pharmacological studies. Ticks Tick Borne Dis. 2025;16 5:102534. 10.1016/j.ttbdis.2025.102534 40789245

[44] Silman I. The multiple biological roles of the cholinesterases. Prog Biophys Mol Biol. 2021;162:41–56. 10.1016/j.pbiomolbio.2020.12.001. 33307019

[45] Paraoanu LE, Layer PG. Acetylcholinesterase in cell adhesion, neurite growth and network formation. FEBS J. 2008;275:618–24. 10.1111/j.1742-4658.2007.06237.x. 18205832

[46] Giordani G, Cattabriga G, Becchimanzi A, Di Lelio I, De Leva G, Gigliotti S, et al. Role of neuronal and non-neuronal acetylcholine signaling in *Drosophila* humoral immunity. Insect Biochem Mol Biol. 2023;153:103899. 10.1016/j.ibmb.2022.103899. 36596348

[47] Mashimo M, Moriwaki Y, Misawa H, Kawashima K, Fujii T. Regulation of immune functions by non-neuronal acetylcholine (ACh) via muscarinic and nicotinic ACh receptors. Int J Mol Sci. 2021. 10.3390/ijms22136818. PMC8268711 34202925

[48] Orchard I, Al-Dailami AN, Lange AB. Acetylcholine and Malpighian tubules of the kissing bug, *Rhodnius prolixus*: evidence for expression of the non-neuronal cholinergic system and a diuretic action of acetylcholine. J Insect Physiol. 2025;161:104752. 10.1016/j.jinsphys.2025.104752. 39890016

[49] Temeyer KB, Schlechte KG, Olafson PU, Drolet BS, Tidwell JP, Osbrink WLA, et al. Association of salivary cholinesterase with arthropod vectors of disease. J Med Entomol. 2020;57:1679–85. 10.1093/jme/tjaa096. 32459332

[50] Temeyer KB, Tuckow AP. Tick salivary cholinesterase: a probable immunomodulator of host-parasite interactions. J Med Entomol. 2016;53:500–4. 10.1093/jme/tjv252. 26794231

