# Supplementary material for: Putative acetylcholinesterase genes from a one-host tick species, the winter tick (Dermacentor albipictus)
Source: Parasit Vectors. 2026 Feb 14;19:146. doi: 10.1186/s13071-025-07205-z (PMC13625402; doi:10.1186/s13071-025-07205-z)
Supplement: Supplementary file 1 [file 13071_2025_7205_MOESM3_ESM.docx]

**Percent Identity Matrices**

*Rhipicephalus sanguineus* homologoues of AChE duplicated set

```
			   1       2.      3.      4.     5.      6.      7
1: Rsang_XP_037517045.1  100.00   42.75   39.90   50.10   47.50   50.93   49.49
2: Rsang_XP_037515686.1   42.75  100.00   83.13   49.60   46.10   50.30   47.15
3: Rsang_XP_037517848.1   39.90   83.13  100.00   48.16   43.24   49.39   46.00
4: Rsang_XP_037517835.1   50.10   49.60   48.16  100.00   57.91   64.61   61.87
5: Rsang_XP_037517832.1   47.50   46.10   43.24   57.91  100.00   78.86   80.40
6: Rsang_XP_037517833.1   50.93   50.30   49.39   64.61   78.86  100.00   88.22
7: Rsang_XP_037517834.1   49.49   47.15   46.00   61.87   80.40   88.22  100.00
```

*Dermacentor andersoni* homologoues of AChE duplicated set

```
			 1	 2	 3	4       5	6
1: Dand_XP_050034532.1  100.00   61.60   47.26   49.16   50.56   52.22
2: Dand_XP_050034533.2   61.60  100.00   48.45   49.69   50.11   50.93
3: Dand_XP_050035422.2   47.26   48.45  100.00   60.24   50.12   48.07
4: Dand_XP_050035829.3   49.16   49.69   60.24  100.00   52.32   49.29
5: Dand_XP_050035314.3   50.56   50.11   50.12   52.32  100.00   55.26
6: Dand_XP_072144491.1   52.22   50.93   48.07   49.29   55.26  100.00
```

*Dermacentor variabilis* homologoues of AChE duplicated set

```
     			 1	  2	 3	4	5       6
1: Dvar_XP_075547911.1  100.00   59.53   47.91   48.30   48.73   47.18
2: Dvar_XP_075550075.1   59.53  100.00   48.67   50.19   49.91   48.18
3: Dvar_XP_075547471.1   47.91   48.67  100.00   60.84   49.81   52.47
4: Dvar_XP_075550192.1   48.30   50.19   60.84  100.00   50.19   50.94
5: Dvar_XP_075547386.1   48.73   49.91   49.81   50.19  100.00   52.92
6: Dvar_XP_075547395.1   47.18   48.18   52.47   50.94   52.92  100.00
```
